# Influence of the non-equal aligned nozzles for fuel injection inside the supersonic combustion chamber

**DOI:** 10.1038/s41598-024-63544-4

**Published:** 2024-06-04

**Authors:** Zhongmian Fan, Lingxiao Wang, Fei Xu, Xiuli Zhang, Baoling Xie, Yushi Wen, He Li, Saman Aminian

**Affiliations:** 1 School of Chemical Equipment, Shenyang University of Technology, Liaoyang, 111003 Liaoning China; 2 Colleges of Engineering, Science and Research Branch, Islamic Azad University, Tehran, Iran

**Keywords:** Combustion chamber, Fuel jet, Non-equal jets, Supersonic flow, Aerospace engineering, Mechanical engineering

## Abstract

The importance of fuel mixing for the progress of the scramjet engine is indisputable. The present article shows the importance of the non-equal multi-injector system for effective fuel distribution and flame holding inside the combustion segment of a scramjet engine. The supersonic air and fuel jet flow in the non-equal nozzle arrangement is simulated via computational fluid dynamic technique. Two injector types of circular and rectangular nozzle have been analyzed to attain flow characteristics of hydrogen jets at supersonic cross flow. Mach contour is also analyzed for these jet arrangements to show the interface of the jet in the non-equal jet arrangement. Besides, addition of internal air jet is also simulated and evaluated in this research. Our investigation shows that the diffusion height of the fuel jet is higher when a rectangular non-equal nozzle is applied. The circular nozzle is more active in the spreading of the fuel in the combustor and the use of an internal air jet effectively increases fuel in a combustor of the scramjet.

## Introduction

Advance in spacecraft and high-speed vehicles has been required for the access to the far distance in space for explorations^1–3^. Thus, different aspects and segments of spacecraft has been investigated by researchers to control and improve the efficiency of this vehicles in far distance^4,5^. The achievements by aerospace scientists have been used in several industrial and commercial applications in recent years^6–8^. Among different segments of spacecrafts, efficient fuel injection at supersonic combustion chambers is pivotal for the optimal performance of propulsion systems. This article explores the significance of non-equal aligned nozzles for fuel injection within these chambers^9,10^. Additionally, it emphasizes the use of computational techniques to analyze flow patterns and shock interactions within the combustion chamber.

Previous studies have highlighted the critical role of fuel injection in supersonic combustion chambers, emphasizing the need for effective mixing and distribution to achieve optimal combustion efficiency^10^. Moreover, research has underscored the impact of nozzle design on fuel injection performance, particularly the advantages of each nozzle types in enhancing fuel distribution and flame stability within the combustion chamber^11^. Furthermore, the integration of computational techniques for flow analysis and shock interaction has been demonstrated as a valuable approach in understanding the complex dynamics of supersonic combustion, thereby contributing to advancements in propulsion system development^12,13^.

In the quest for improved fuel efficiency and reduced emissions, the automotive industry has been continuously exploring innovative technologies^14–16^. One such technology that has gained significant attention is the multi-injector for efficient fuel mixing in internal combustion engines. By using multiple injectors strategically positioned within the engine, fuel mixing can be optimized, results in enhanced combustion efficiency and reduced pollutant emissions^17,18^.

The purpose of a fuel injector is to deliver the precise amount of fuel into the combustion chamber at the right time^16,17^. Traditional fuel injection systems rely on a single injector, which may not always provide the desired fuel–air mixture distribution across the combustion chamber. This non-uniform distribution can result in inefficient combustion, leading to reduced power output, increased fuel consumption, and elevated emissions^18^.

To overcome these limitations, multi-injector systems have been developed^19–21^. Jiang et al.^20^ presents valuable results about influence of trapezoidal lobe strut on fuel mixing and combustion in supersonic combustion chamber. These systems employ multiple injectors, each strategically positioned to ensure thorough fuel–air mixing within the combustion chamber. Sun et al.^21^ disclose mixing efficiency of hydrogen multijet through backward-facing steps at supersonic flow. By distributing the fuel across various locations, multi-injector systems can create a more homogeneous fuel–air mixture, improving combustion efficiency and overall engine performance^22–24^.

The size of the injectors used in multi-injector systems can vary depending on the specific engine requirements. Factors such as engine displacement, power output, and desired fuel flow rate influence the sizing of the injectors. Typically, the size of the injectors is determined based on the desired fuel atomization and spray characteristics, which directly impact the fuel–air mixing process^25,26^.

Hydrogen exhibits several important flow characteristics when it used as a fuel in a supersonic combustion chamber. Hydrogen is a light gas with low density, which contributes to faster mixing with air and rapid combustion processes in the supersonic combustion chamber and these flow characteristics of hydrogen make it a favorable choice for use in supersonic combustion chambers^23^.

Computational Fluid Dynamics (CFD) has a critical role in the design and optimization of multi-injector systems^27–29^. CFD simulations enable engineers to analyze the fuel flow patterns, spray dispersion, and combustion characteristics within the combustion chamber. By iteratively refining the design and injector sizes through CFD simulations, engineers can achieve an optimal fuel–air mixture and combustion process, leading to better engine performance and reduced emissions^30–32^.

In conclusion, the usage of multi-injector systems for efficient fuel mixing in internal combustion engines offers significant benefits. By employing multiple injectors strategically positioned within the combustion chamber, these systems enhance fuel–air mixing, resulting in developed combustion efficiency^33–35^. The sizing of the injectors is determined based on engine requirements, and CFD simulations play a vital role in optimizing the design and performance of multi-injector systems.

Efficient fuel mixing is paramount in optimizing combustion processes within internal combustion engines, gas turbines, and various industrial applications. The use of multi-injectors has emerged as a promising strategy to increase fuel–air mixing and enhanced performance. CFD plays a crucial role in analyzing the performance of multi-injectors by simulating the complex fluid dynamics involved in the mixing process. One critical parameter in multi-injector design is the size of the injector, which significantly influences fuel atomization and distribution within the combustion chamber^34,35^.

The size of the injector in multi-injector systems refers to the diameter and configuration of the orifices through which fuel is released into the combustion chamber. This dimension directly affects the atomization and dispersion characteristics of the fuel spray, influencing its interaction with the surrounding air and subsequent combustion behavior^31,36^. Proper sizing of injectors is essential to achieve optimal fuel distribution, avoid fuel impingement on chamber walls, and minimize the formation of undesirable combustion byproducts such as unburned hydrocarbons and particulate matter. The diffusion mechanism of the hydrogen for efficient fuel mixing has been investigated in previous published articles^34,37^. These papers reveal the importance of the hydrogen gas diffusion when encounter transverse supersonic flow. Meanwhile, the interaction of the fuel jet has also addressed in these works.

In this context, understanding the power of injector size on fuel mixing efficiency through CFD simulations becomes indispensable. By employing advanced numerical modeling techniques, engineers can analyze the fluid dynamics inside combustion chambers with varying injector sizes, providing insights into flow patterns, fuel dispersion, and combustion characteristics. Such simulations enable the optimization of injector dimensions to achieve the desired fuel–air mixing quality^37,38^. Abdollahi et al.^37^ investigated influence of extruded injector nozzle on fuel mixing and mass diffusion of multi fuel jets in the supersonic cross flow via computational study.

This paper explores the usage of multi-injectors for efficient fuel mixing, with a specific focus on the significance of injector size in influencing combustion performance. Through a combination of theoretical analysis and CFD simulations, the effects of injector dimensions on fuel atomization, spray characteristics, and combustion efficiency are investigated. Additionally, practical implications for the design and optimization of multi-injector systems in automotive, aerospace, and industrial applications are discussed. Although several papers have been focused on multi-jet system for fuel mixing in the combustor, the influence of the non-equal nozzles has not investigated in the past works. Besides, the combination of the coaxial and annular system with non-equal nozzles has not been investigated via three-dimensional modeling.

The role of the non-equal injectors for efficient fuel mixing in transverse fuel injection systems has been investigated in the present study. A computational study has been done to reveal the interactions of fuel jets when the size of the nozzle is not equal. The role of nozzle types (circular and rectangular) on fuel diffusion is also compared in this research paper. This study also demonstrates the fuel concentrations and mixing on the jet plane for both annular and coaxial fuel injection at a supersonic combustion chamber. Flow analysis is also performed to compare the performance of the proposed system with the conventional equal jet system.

## Governing equations and numerical methodology

The modeling of fuel mixing of the transverse jet at the high-velocity free stream at the combustion chamber is mainly done via solving Reynolds-averaged Navier–Stokes equation (RANS) equations^37,38^. The supersonic inflow encounters the fuel jet and the cross-jet flow creates the shock wave near the jet consequently, the energy equation is also coupled for the existence of the shock wave. The flow inside the model is presumed ideal gas and the turbulence model of Shear Stress Transport (SST) is applied for the modeling of flow through the combustor^33,37,41,42^. The simulation of the injection of secondary gas requires a species transport equation and this study has used this equation for the simulation of the hydrogen cross-jet flow in the supersonic air stream. The details of the main governing equation are available in the previously published papers^10,17,36^.

**Figure 1:**
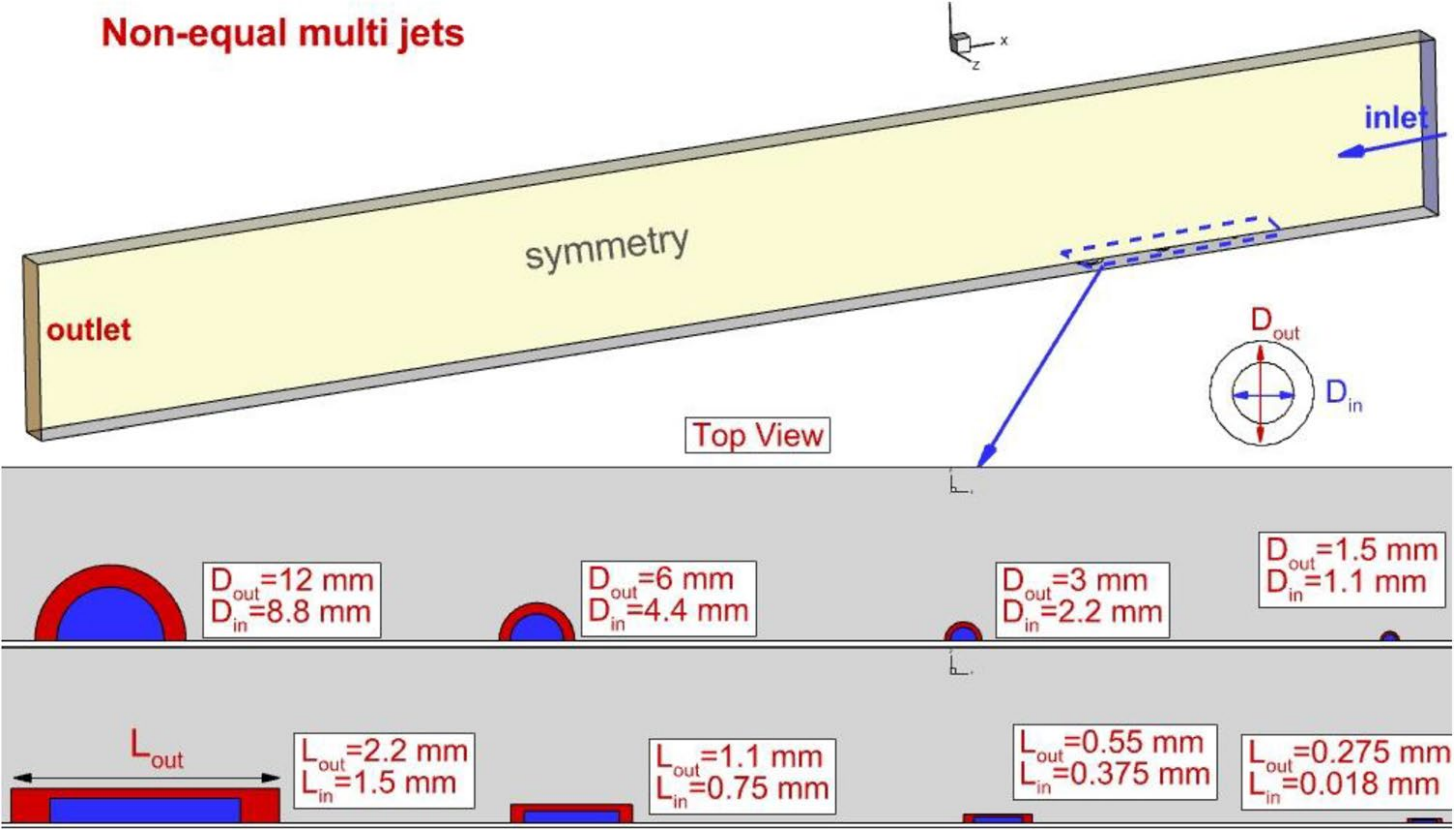
The details of the non-equal multi-injectors.

Figure 1 exemplifies the size and geometry of the projected injection system of multi non-equal nozzle in the combustion chamber. The figure displays the annular rectangular and circular four jets located in the combustor. The symmetry of the domain is chosen for the simulation and the length, height, and width of the domain is 110 mm, 10 mm, and 4 mm, respectively. The size of the inner and outer nozzles is displayed in Fig. 1. L_out_ and L_in_ in the rectangular nozzle represents for length of outer and inner rectangular, respectively. The first nozzle is positioned 20 mm behind the inlet while the last one is located 14 mm behind the first nozzle. The free stream velocity is equivalent to Mach = 4 at atmospheric pressure and temperature of 1000 K. The concept of the nozzle changes is based on the doubling of the surface area of the nozzle upstream as presented in Fig. 1. The total pressure of a fuel jet is 27% of frees stream total pressure which is equal to stochastic H2 combustion in a scramjet engine. Besides, the surface area of the annular and internal nozzle is identical. Meanwhile, the total surface area of these four non-equal circular and rectangular fuel injectors is equivalent to four equal nozzles with a diameter of 0.5 mm.

**Figure 2:**
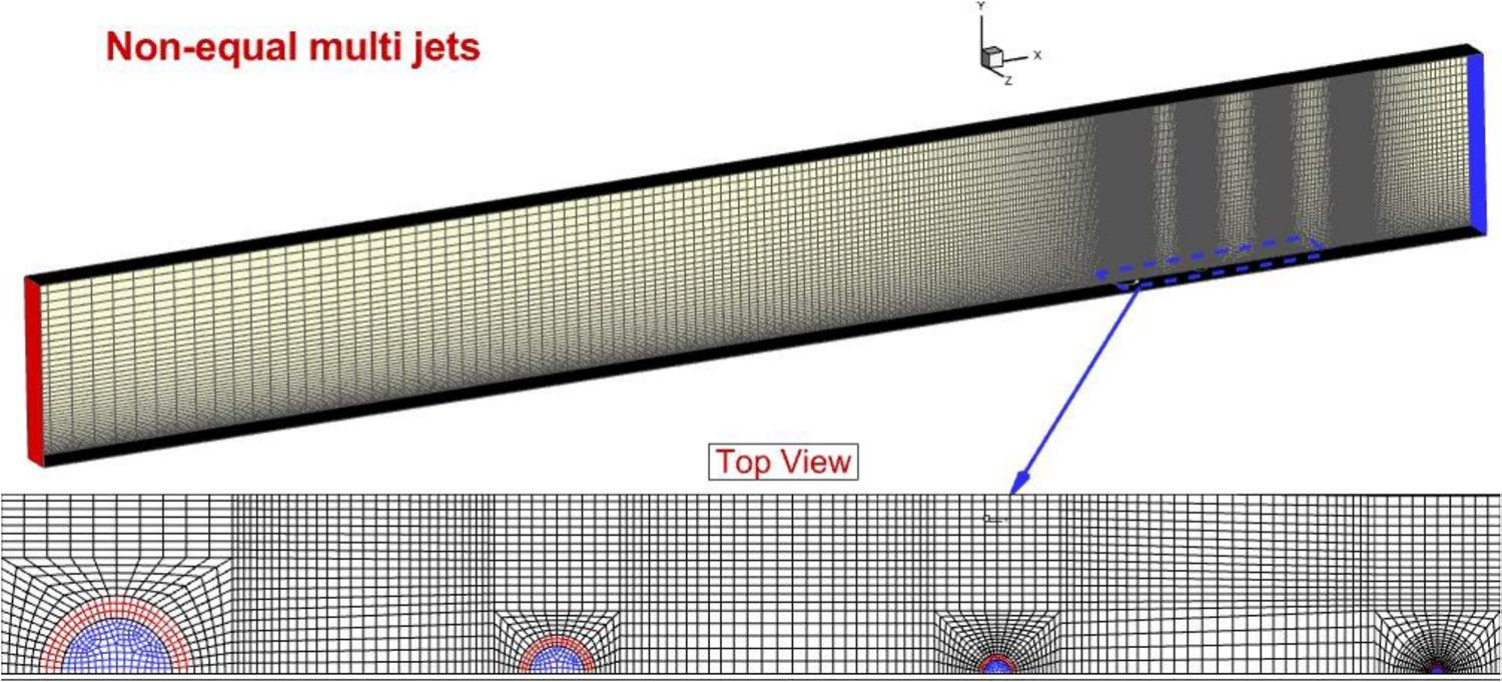
Grid production.

The generated grid for the present model is exhibited in Fig. 2. The size of the structured grid for the model is based on the flow change and importance of the region. The region like the injector area in which flow interactions are expected has higher grid resolution than other sections. The size of the grid is enlarged along the height of the domain since the main important section of the jet flow and mixing occurs near the bottom. The grid analyses are also performed to authenticate that the selected grid is sufficient for the modeling of the proposed injection models. Table 1 displays the details of the created grids and the fine grid is selected for our investigation.

**Table 1:** Grid study analysis.

	Cells	Grid cells. along X, Y and Z direction	Average fuel concentration at 11 mm of cavity
Coarse	640,000	320 × 100 × 20	0.162
medium	1,590,000	380 × 140 × 30	0.181
fine	2,856,000	420 × 170 × 40	0.187
Very fine	4,800,100	480 × 200 × 50	0.188

## Results and discussion

The assessment of the present simulation with experimental data is done and presented in Table 2. The selected model for the verification is a single circular nozzle with a diameter of 2 mm at a supersonic flow of Mach = 4. The size of the penetration height on the jet plane is equated with other computational and experimental methods^41,42^. The evaluation of the results shows that the applied computational methodology and chosen governing equation are reasonable and rational for the proposed problem.

**Table 2:** Validation of penetration height.

Downstream (mm)	Numerical data of Pudsey et al. (mm)^42^	Present simulation (mm)
10	8.0	7.78
20	8.5	8.32
30	8.6	8.58
40	9.6	9.45
50	9.9	9.66

**Figure 3:**
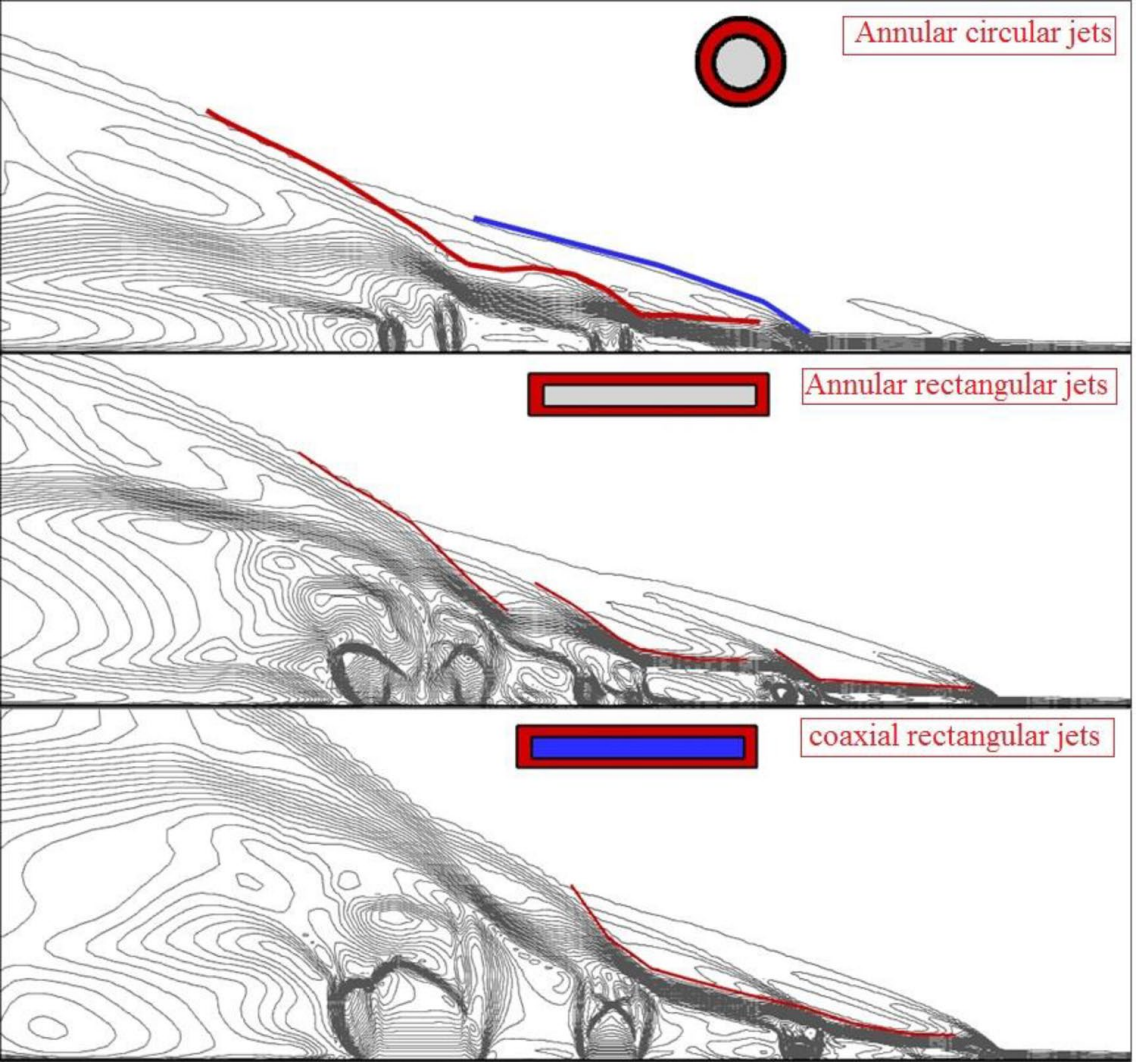
Mach contour on a jet plane.

Figure 3 displays the contour of the Mach on the jet plane for three fuel injector configurations circular nozzle, rectangular nozzle, and coaxial rectangular nozzle. In the circular nozzle, the interactions of the hydrogen jet on the symmetry plane are limited in comparison with the rectangular nozzle since more segments of the fuel are released on the jet plane in the rectangular nozzle. Since the fuel is released from the annular region, more effects in the distance of the domain are observed in the circular jet. However, the jet nozzle has more intense interactions in the rectangular injector configuration. In the figure, the blue line and red line stand for the bow shock and shear layer that is created via the interaction of the fuel jet and free stream. The produced barrel shock near the jet also demonstrates the main impacts of nozzle type on the flow near the nozzle. Addition of the inward air jet for efficient fuel mixing indicates that the produced barrel shock varies momentously in our model. As displayed in the figure, the shape of the barrel shock confirms the higher expansion of the fuel jet by usage of the inner air jet. The slant of the shear layer (red line) is also higher by the injection of the internal air jet.

**Figure 4:**
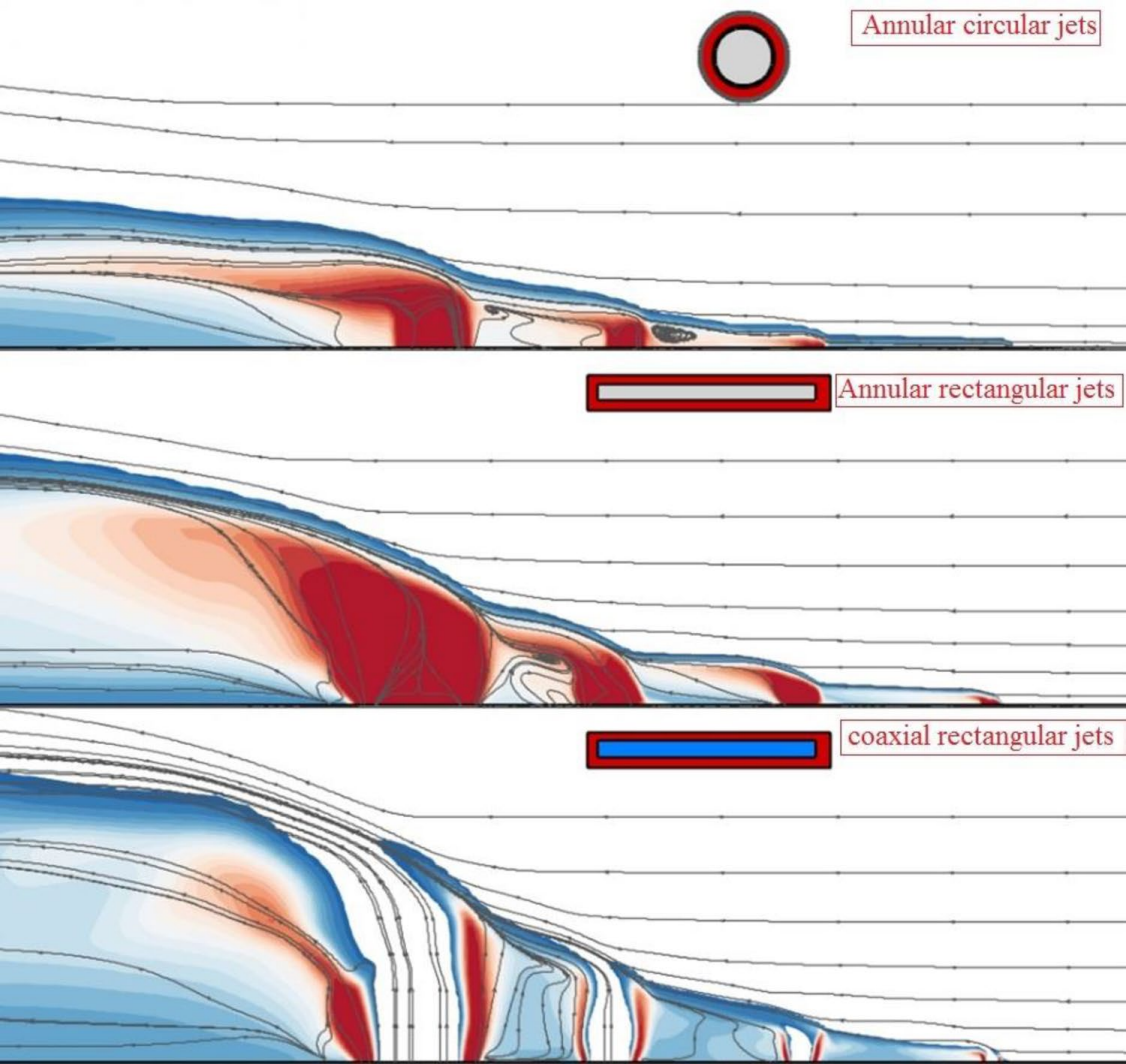
Mixing zone and flow stream on the jet plane.

The fuel mixing zone behind the fuel jet in three different configurations has been compared in Fig. 4. The comparison of the annular configuration of circular and rectangular jets indicates that the fuel penetration height is higher in rectangular systems. The addition of the internal air jet also extends the height of the fuel jet while fuel concentration is more homogeny. The deflection of the free stream air jet has been increased while the air jet is free from the internal nozzle.

The coaxial injection system in the combustion chamber of a scramjet engine offers several advantages, promoting efficient combustion and enhancing overall engine performance^31,34^. The coaxial injection system is typically more compact than other injection methods, saving space and weight in the engine system. The coaxial configuration helps maintain stable combustion by ensuring uniform fuel distribution and ignition within the chamber^37^.

**Figure 5:**
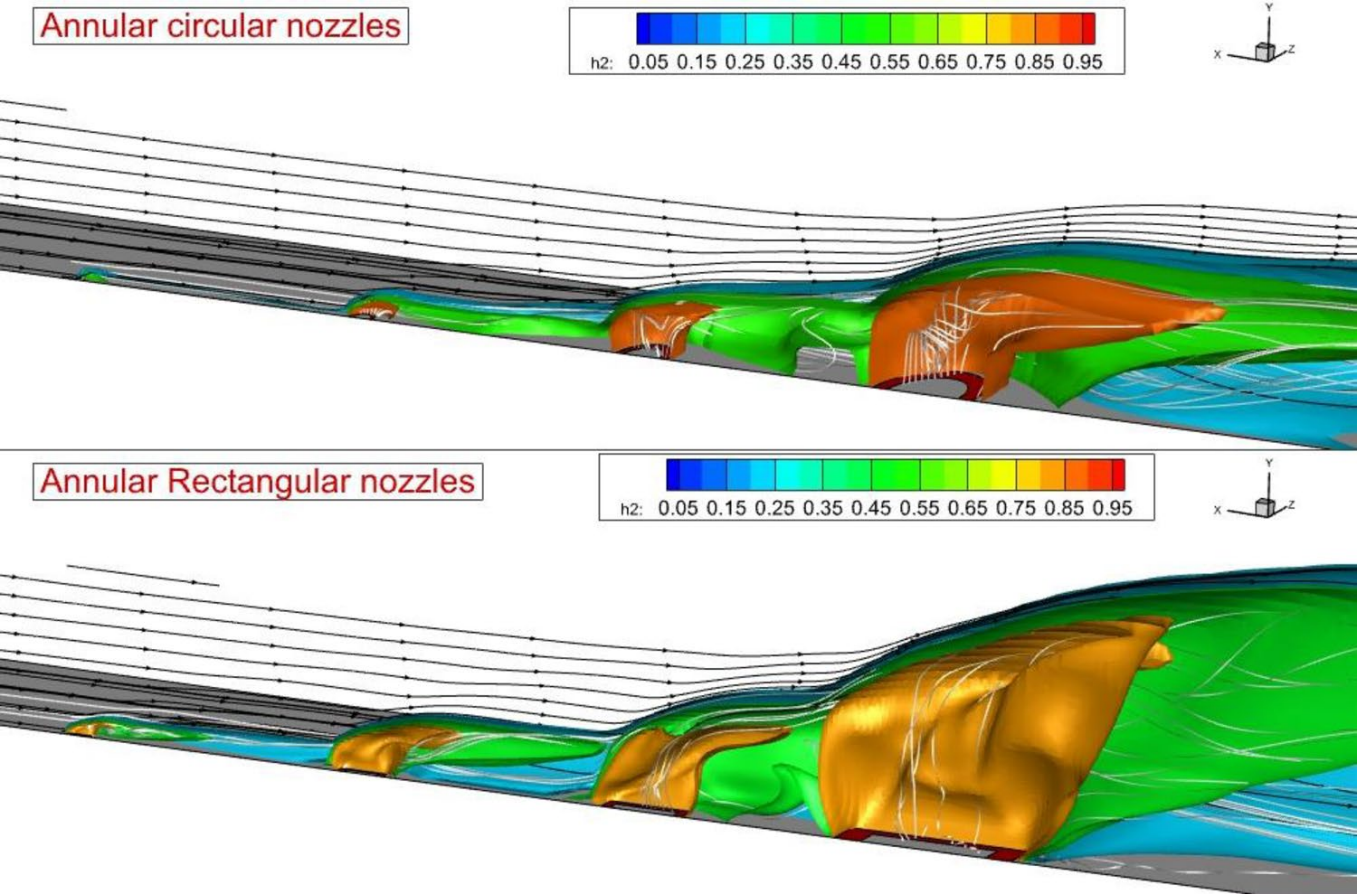
3-D fuel jet structure and air stream of circular and rectangular nozzles.

**Figure 6:**
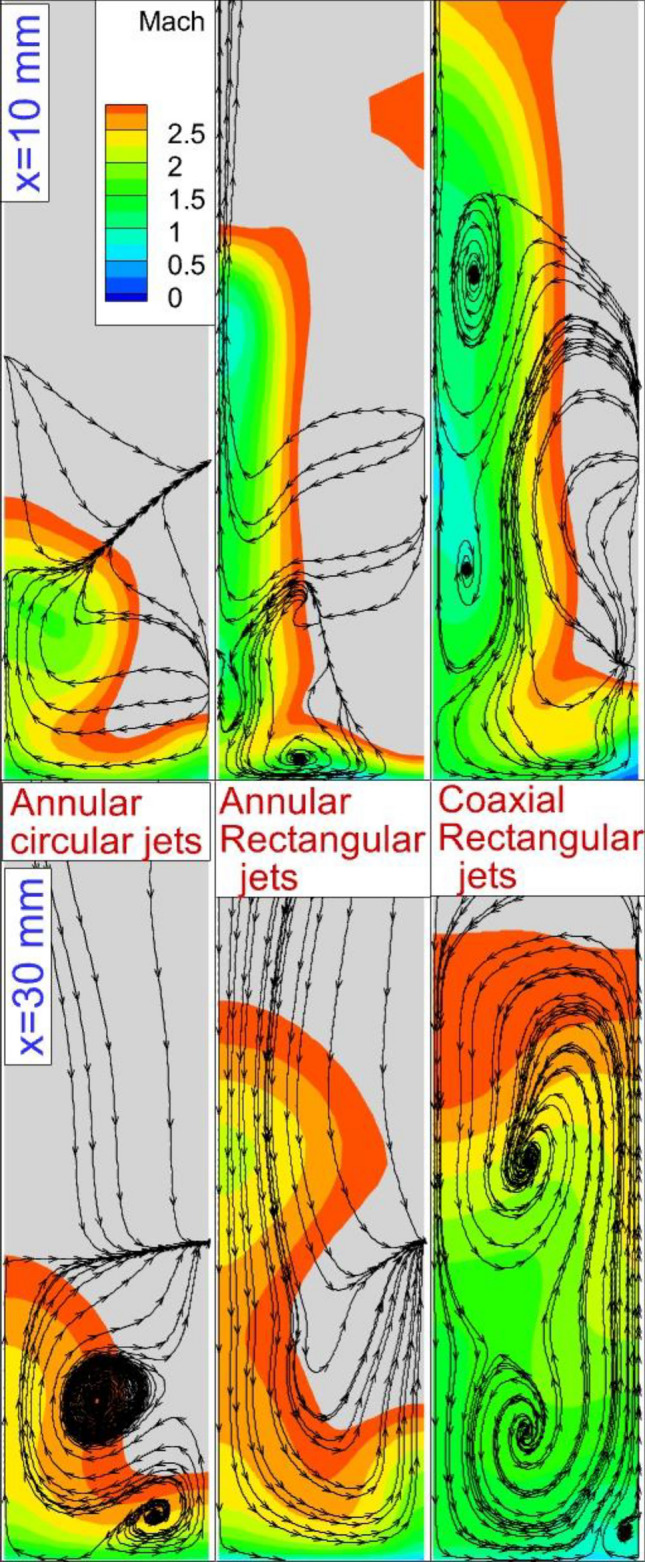
Mach contour and flow stream on two planes behind nozzles.

**Figure 7:**
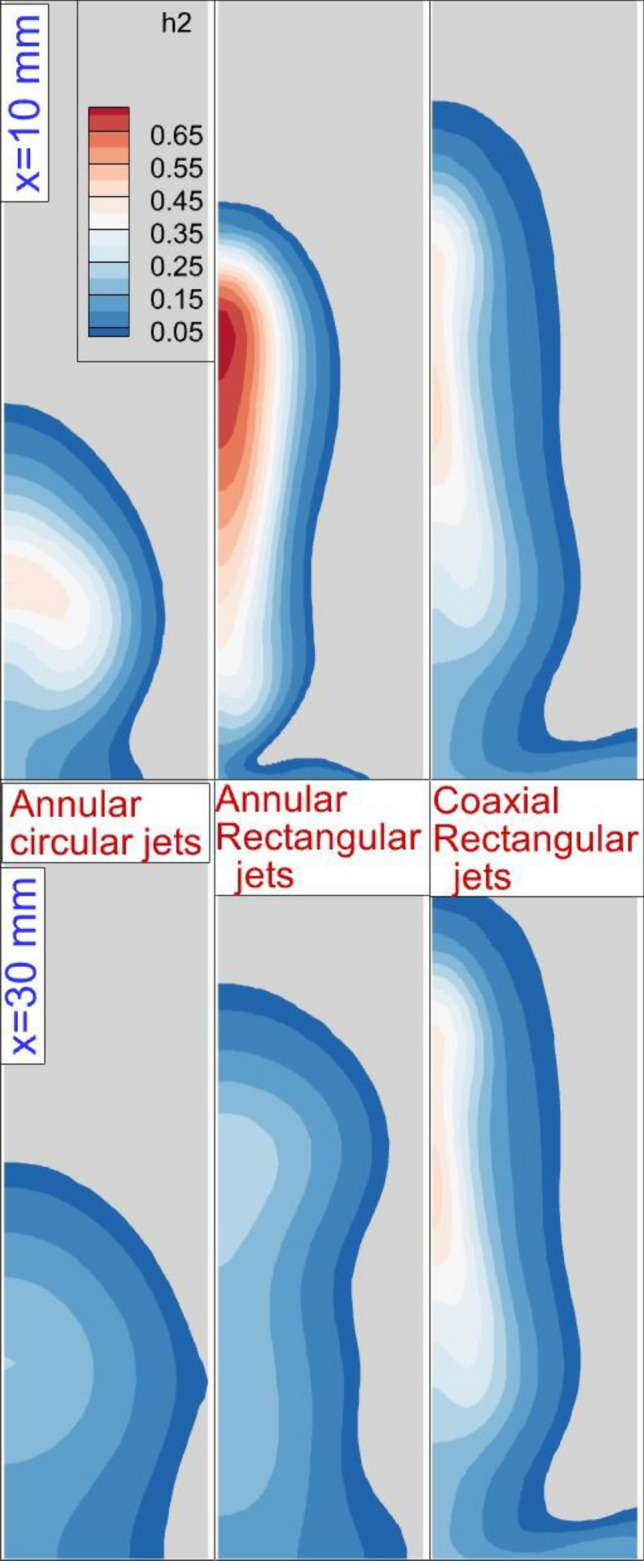
The mixing zone on two planes behind nozzles.

Figure 5 visualizes the 3-D feature of the annular fuel jet free from rectangular and circular nozzles. The feature of the annular jet considerably changes as the injector type is changed. A comparison of the nozzle-type effects reveals that the height of the jet plume is increased as the rectangular injector is applied. This finding also indicates that the nozzle that distributes along the flow has a more expansive fuel jet plume. The deflection of the air stream is more as the rectangular injector is applied. The ratio of the rectangular nozzle is also an important factor for efficient fuel mixing. Figure 6 displays the Mach contour on the two planes located behind the different injectors. The figure also presents the flow stream on these planes. The changes in the Mach contour visibly illustrate the development of the vortex pair behind the jet. The injection of the air stream from the core of the annular rectangular nozzle also improves the fuel jet penetration behind the injector. On the second plane (x = 30 mm), the jet vortex pair expands and the fuel jet is improved more. The fuel mixing zone behind the injector is illustrated in Fig. 7. The fuel distribution of rectangular and circular injectors could be compared and it is noticed that the concentration of the fuel released from the rectangular nozzle is along the height of the combustor while the circular nozzle improves the fuel jet along the width of the combustor. In the coaxial rectangular jet configuration, the height of the nozzle is improved as the vortex pair arrangement is amplified. Although the concentration of the fuel is more homogeny far downstream, the arrangement of the fuel jet is preserved in this region.

**Figure 8:**
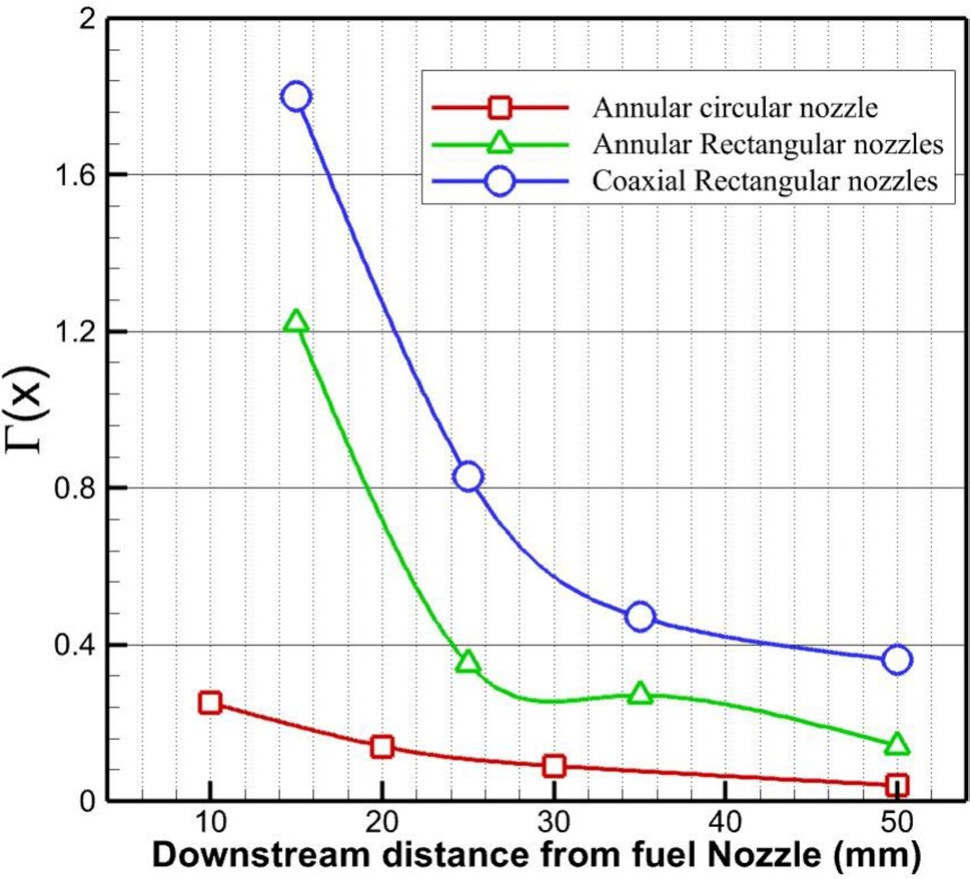
Impact of the jet configuration on strength of the circulation.

The circulation strength of these three jet configurations downstream of the injectors is compared in Fig. 8. The change in the circulation power shows that the rectangular jet has a more powerful circulation downstream of the injectors. The meaningful difference of rectangular injection is related to the higher interaction of the fuel on the jet plane and the formation of the more powerful circulation in the gap of the injector. Meanwhile, the jet-induced circulation strength is improved as the air is applied from the internal nozzle. As explained before, the strength of the vortices in the distance of the jet is augmented by the air injection. The considerable drop in the circulation strength may be associated with the considerable size of the last injector. The formulation for calculation of the circulation is as follows:

1 $$\Gamma (x)=\frac{1}{{d}_{j}^{*}{u}_{i}}\iint \left|\frac{\partial v}{\partial z}-\frac{\partial w}{\partial y}\right|\text{d}A$$

**Figure 9:**
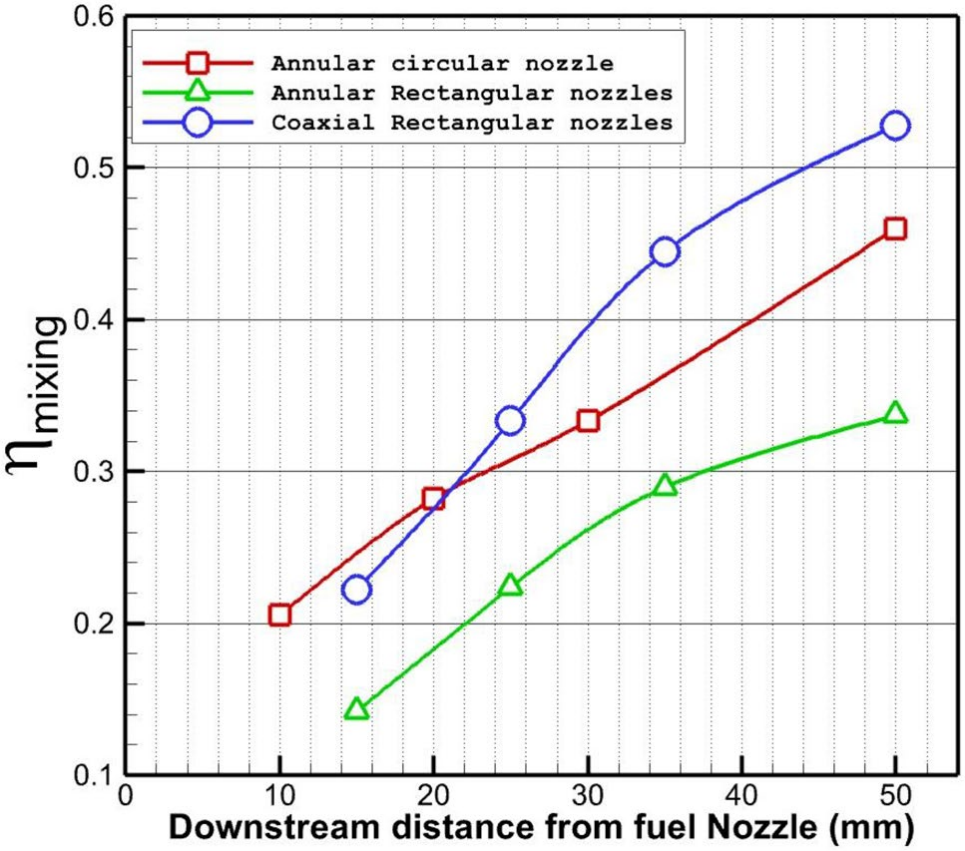
Comparison of fuel mixing of the non-equal injector behind the injector.

The fuel mixing efficacy downstream of the three introduced injector configurations is compared in Fig. 9. The evaluation of the mixing efficacy behind the injector specifies that fuel mixing factor of the circular nozzle is more efficient than rectangular injector near behind the nozzle. Besides, the addition of the internal flow of the air jet has sizably increased the mixing of the fuel behind the injector. More than a 55% increase in the fuel mixing of the rectangular injector is perceived by the injection of an internal sonic air jet. Lee^42^ offered the definition for the calculation of mixing as follows:

2 $$\eta_{mix} = {\raise0.7ex\hbox{${\iint {Y_{{H_{2} }}^{r} }\rho u.dy.dz}$} \!\mathord{\left/ {\vphantom {{\iint {Y_{{H_{2} }}^{r} }\rho u.dy.dz} {\iint {Y_{{H_{2} }}^{{}} }\rho u.dy.dz}}}\right.\kern-0pt} \!\lower0.7ex\hbox{${\iint {Y_{{H_{2} }}^{{}} }\rho u.dy.dz}$}}$$

where

3 $$Y_{{H_{2} }}^{r} = \left\{ \begin{gathered} Y_{{H_{2} }}^{{}} ,Y_{{H_{2} }}^{{}} \le Y_{{H_{2} }}^{st} \hfill \\ Y_{{H_{2} }}^{st} (\frac{{1 - Y_{{H_{2} }}^{{}} }}{{1 - Y_{{H_{2} }}^{st} }}),Y_{{H_{2} }}^{{}} > Y_{{H_{2} }}^{st} \hfill \\ \end{gathered} \right.$$

where $Y_{{H_{2} }}^{st}$ is the stoichiometric hydrogen concentration for a fuel/air mixture.

## Conclusion

The computational method is employed to compare the efficient fuel injection released from multi-non-equal nozzles inside the combustor of scramjet engine. Two annular injector types (rectangular and circular) are investigated and the fuel jet characteristics are analyzed via evaluation of the shock wave interactions. The usage of an internal air jet for the development of a non-equal multi-jet is entirely inspected to reveal the role of the proposed nozzle arrangement on the distribution of hydrogen along the combustor. The power of circulation as well as the performance of the fuel mixing in the non-equal injectors have been investigated in detail. The evaluation of the fuel penetration approves that the fuel height penetration is more efficient in the rectangular non-equal injectors. Fuel mixing analyses behind the non-equal injectors show that fuel mixing is enhanced more via a circular nozzle. The fuel mixing via the rectangular injector is enhanced when the internal air jet is applied. The use of rectangular non-equal injectors in supersonic combustion chambers has been shown to enhance fuel penetration efficiency compared to other injector geometries. This design allows for better fuel distribution and penetration into the combustion chamber, improving mixing with the incoming air and combustion efficiency. The increased fuel penetration efficiency in rectangular non-equal injectors results in more effective fuel–air mixing, leading to enhanced combustion performance and overall engine efficiency.

## Data Availability

All data generated or analysed during this study are included in this published article.
